# No evidence of response bias in a population-based childhood cancer survivor questionnaire survey — Results from the Swiss Childhood Cancer Survivor Study

**DOI:** 10.1371/journal.pone.0176442

**Published:** 2017-05-02

**Authors:** Corina S. Rueegg, Micòl E. Gianinazzi, Gisela Michel, Marcel Zwahlen, Nicolas X. von der Weid, Claudia E. Kuehni, and the Swiss Paediatric Oncology Group (SPOG)

**Affiliations:** 1 Swiss Childhood Cancer Registry, Institute of Social and Preventive Medicine, University of Bern, Bern, Switzerland; 2 Oslo Centre for Biostatistics and Epidemiology, Department of Biostatistics, Institute of Basic Medical Sciences, University of Oslo, Oslo, Norway; 3 Department of Health Sciences and Health Policy, University of Lucerne, Lucerne, Switzerland; 4 Paediatric Hematology/Oncology Unit, University Children’s Hospital Basel (UKBB), University of Basel, Basel, Switzerland; 5 Paediatric Epidemiology, Children’s University Hospital of Bern, University of Bern, Bern, Switzerland; Leibniz Institute for Prvention Research and Epidemiology BIPS, GERMANY

## Abstract

**Purpose:**

This is the first study to quantify potential nonresponse bias in a childhood cancer survivor questionnaire survey. We describe early and late responders and nonresponders, and estimate nonresponse bias in a nationwide questionnaire survey of survivors.

**Methods:**

In the Swiss Childhood Cancer Survivor Study, we compared characteristics of early responders (who answered an initial questionnaire), late responders (who answered after ≥1 reminder) and nonresponders. Sociodemographic and cancer-related information was available for the whole population from the Swiss Childhood Cancer Registry. We compared observed prevalence of typical outcomes in responders to the expected prevalence in a complete (100% response) representative population we constructed in order to estimate the effect of nonresponse bias. We constructed the complete population using inverse probability of participation weights.

**Results:**

Of 2328 survivors, 930 returned the initial questionnaire (40%); 671 returned the questionnaire after ≥1reminder (29%). Compared to early and late responders, we found that the 727 nonresponders (31%) were more likely male, aged <20 years, French or Italian speaking, of foreign nationality, diagnosed with lymphoma or a CNS or germ cell tumor, and treated only with surgery. But observed prevalence of typical estimates (somatic health, medical care, mental health, health behaviors) was similar among the sample of early responders (40%), all responders (69%), and the complete representative population (100%). In this survey, nonresponse bias did not seem to influence observed prevalence estimates.

**Conclusion:**

Nonresponse bias may play only a minor role in childhood cancer survivor studies, suggesting that results can be generalized to the whole population of such cancer survivors and applied in clinical practice.

## Introduction

Nonresponse bias can affect inferences drawn from questionnaire surveys across different populations, countries, and topics [1–6]. A decrease in response rates to questionnaire surveys over the past 30 years may have increased the extent of bias and aggravated this problem [5, 7–9]. In studies of general population samples in North America and Europe, nonresponders were more often male, less educated, single or divorced, childless, or immigrants [5–7, 10, 11]. This underrepresentation of certain subpopulations in surveys could lead to an under- or overestimation of effects and estimates in results [1–6]. The biased results might then lead to flawed decisions. For example, the relevance of psychological late effects in childhood cancer survivors might be under-recognized, resulting in insufficient mental health follow-up care.

To avoid nonresponse bias, researchers try to increase the number of responders in their surveys by reminding nonresponders in different ways. However, only a few studies, using telephone surveys in the general population, have compared the characteristics of early responders to late responders, who responded only after being reminded, to determine whether the reminder calls affected prevalence estimates [9, 12]. They found that the enhanced calling efforts increased the response rate in the survey. However, they found very little, nonsignificant change in their results by including interviews with persons from whom it had been more difficult to elicit responses.

Several large questionnaire surveys in Switzerland, the United States, Canada, and the United Kingdom have investigated long-term outcomes of childhood cancer. Their response rates vary, ranging between 63 and 79% [13–16], but no study has investigated nonresponse bias. Understanding such (potential) bias would be particularly important for this population because results inform clinical practice and influence treatment of current patients and follow-up care of former patients.

Our study describes characteristics of early responders, late responders, and nonresponders, and estimates the effect of potential nonresponse bias on selected prevalence estimates in this survey. To conduct our analysis, we used a comprehensive dataset of the population-based Swiss Childhood Cancer Registry (SCCR), which contains sociodemographic and cancer-related information for both responders and nonresponders of the Swiss Childhood Cancer Survivor Study (SCCSS).

## Materials and methods

### The Swiss Childhood Cancer Survivor Study

The SCCSS is a population-based, long-term follow-up study of all childhood cancer patients registered in the SCCR who were diagnosed 1976–2005 at age 0–15 years and survived ≥5 years [14]. The SCCR includes all children and adolescents in Switzerland diagnosed before age 21 with leukemia, lymphoma, central nervous system (CNS) tumors, and malignant solid tumors according to the International Classifications of Childhood Cancer (third edition, ICCC-3), or Langerhans cell histiocytosis [17–19]. This analysis included all survivors aged ≥16 years at time of survey.

During the years 2007–2011, 2631 eligible study participants were traced with an extensive address search procedure. Those with valid address received an information letter from their former pediatric oncology clinic, followed by a paper-based questionnaire with a prepaid return envelope. Nonresponders received a reminder letter and another questionnaire 4–6 weeks later. If they did still not respond, they were reminded by phone 4–6 weeks later. Letters and questionnaires were supplied in three national languages: German, French, and Italian. The SCCSS questionnaire is derived from the US and UK childhood cancer survivor studies [13, 15], with some modifications, and covers quality of life, cancer history, somatic health, fertility, current medication, health service utilization, mental health, health behaviors, and socioeconomic information. The questionnaire is published in the three original languages on the homepage of the SCCR (German: http://www.kinderkrebsregister.ch/index.php?id=3709; French: http://www.registretumeursenfants.ch/index.php?id=3859; Italian: http://www.registrotumoripediatrici.ch/index.php?id=3917). Ethics approval was granted through the Ethics Committee of the Canton of Bern to the SCCR and SCCSS (KEK-BE: 166/2014).

### Outcome measure: Response to the questionnaire

We classified response to the questionnaire into three categories: *early responders* (survivors who answered to the initial questionnaire sent), *late responders* (survivors who answered to the first or second reminder), and *nonresponders*.

### Available information on responders and nonresponders

We used information from the Swiss Childhood Cancer Registry to compare those who responded to the questionnaire with those who did not. We compared gender, language (*German*, *French*, or *Italian*), nationality, age at diagnosis, cancer diagnosis, cancer treatment, relapse status, time since diagnosis, and current age. We categorized the migration background of our population into three nationalities according to the languages in which we provided our questionnaires and letters: no migration background (*Swiss*); migration background, but a mother tongue of a language provided in our questionnaire (*German*, *Austrian*, *French*, *or Italian nationality*); migration background lacking mother tongue of a questionnaire language (*other foreigner*).

We also linked our data to the Swiss Neighborhood Index of Socio-Economic Position (Swiss-SEP)[20] to gain census-based, neighborhood-level information on socioeconomic position for the whole population based on the contact address at the time of the survey. We divided the Swiss-SEP into tertiles.

### Statistical analysis

Analyses were performed with Stata, version 12.0 (Stata Corporation, Texas). First, we determined the proportion of early and late responders and nonresponders, and described their characteristics. Second, we determined factors that predict the likelihood (propensity) of response, using univariable and multivariable multinomial regression models; the response status was the outcome of the analysis with being a nonresponder as baseline. Factors associated with the response status at a significance level of p≤0.05 in the univariable regression were kept in the multivariable model.

Third, we used inverse probability of participation weights (**S1 File**) [8, 21], derived from a logistic regression, to construct a population representative of the total eligible population of the SCCSS in its marginal distribution of age, gender, language region, nationality, type of cancer, and relapse status. We pooled early responders and late responders to predict the binary outcome “responder” vs. “nonresponder” for this logistic regression. We applied this propensity score to calculate prevalence estimates and simulate the total population with 100% response.

Finally, we used this total population to estimate the potential impact of nonresponse bias on our results and appraised the gain of additional recruitment efforts to increase the response rate. To do this we compared the prevalence of typical outcomes for each section of the questionnaire (somatic health, medical care, mental health, and health behaviors; S1 Table) between early responders (40% response), all responders (69% response) and the total population obtained from the weighted analysis (100% response). We made a priori choices of outcomes from the questionnaire that 1) were representative of each section of the questionnaire, 2) we had already published or intended to publish, and 3) were reasonably classifiable into a binary variable (S1 Table).

## Results

### Study population

**Fig 1:**
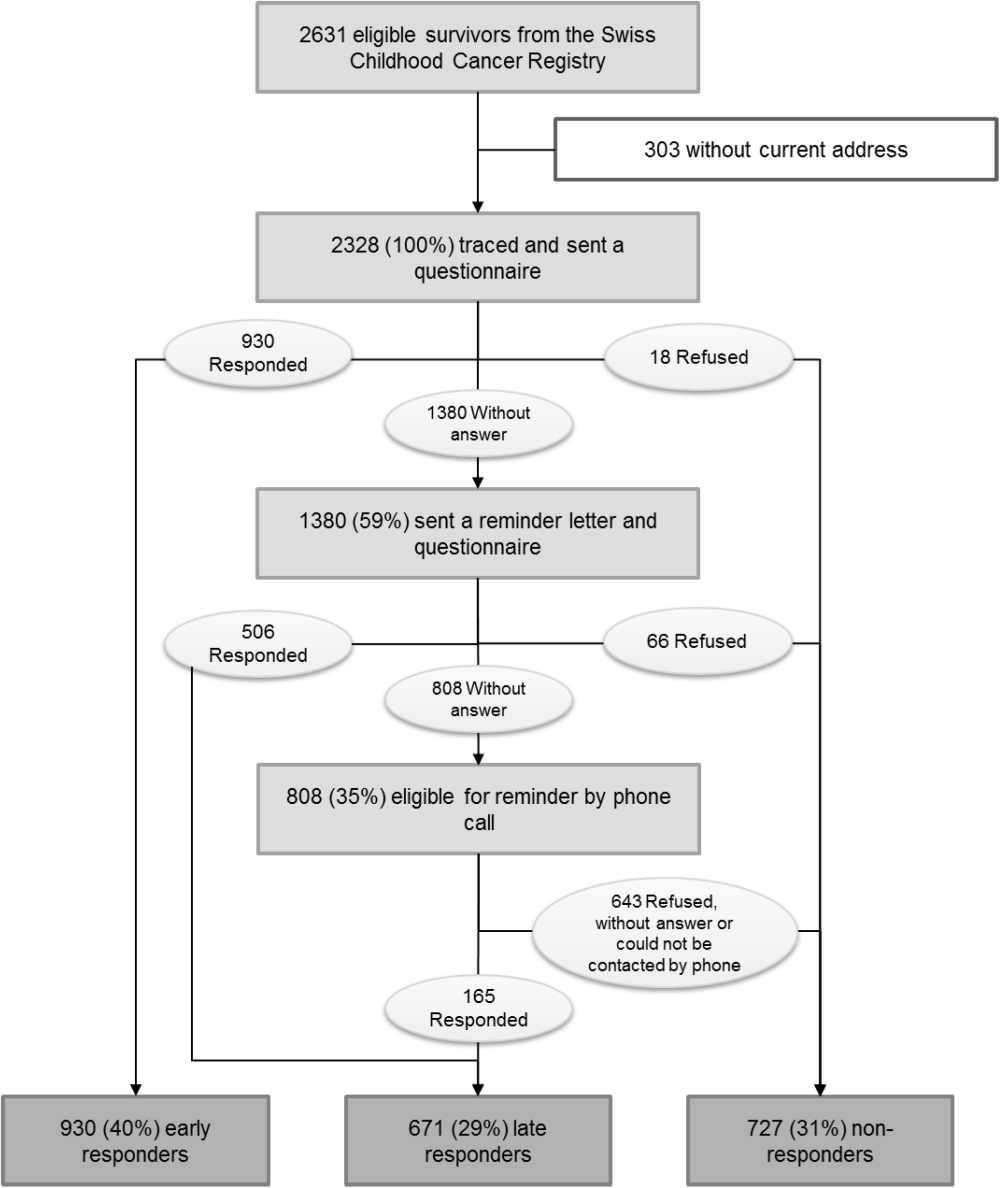
Study design and response behavior in the Swiss Childhood Cancer Survivor Study. The different procedures of the Swiss Childhood Cancer Survivor Study and the number of participants in each step of the study.

We located the addresses of 2328 of 2631 eligible survivors (Fig 1). Of those we found, 930 returned the initial questionnaire (40% of 2328) and 18 declined to participate. Four to six weeks after the first mailing we again sent a questionnaire and a reminder letter to the 1380 survivors who had not yet responded. A further 506 survivors returned the questionnaire (22% of 2328) and 66 declined to participate. Four to six weeks after the second mailing we called the remaining survivors for whom a telephone number was available, and 165 (7% of 2328) returned a questionnaire. Therefore, among the 2328 survivors we located 930 were early responders (40%), 671 were late responders (29%), and 727 who either never responded or explicitly declined participation were characterized as nonresponders (31%). Fig 1 diagrams the procedure by these three groups of survivors obtained from the Swiss Childhood Cancer Registry.

### Characteristics of early, late, and nonresponders

As shown in Table 1, compared to *early* and *late* responders the *nonresponders* more often were male, less than 20 years old, French or Italian speaking, of foreign nationality, diagnosed with a CNS tumor, retinoblastoma, germ cell tumor, or a Langerhans cell histiocytosis, had only had surgical treatment, and were diagnosed less than 10 years ago. For most but not all of the characteristics that were studied, late responders fell between early responders and nonresponders. The associations (risk ratios) between predictors and the likelihood of response were similar across the univariable model (Table 1) and the multivariable model (S2 Table). However, some factors did not reach statistical significance anymore in the multivariable model because of small numbers in the strata language region, type of diagnosis, type of treatment, and time since diagnosis.

**Table 1:** Characteristics of survivors by type of response; risk ratios from univariable multinomial logistic regression models.

		Total (n = 2328)		Early responders^a^ (n = 930)		Late responders^b^ (n = 671)		Non-responders^c^ (n = 727)		RR^f^ early responders	95% CI	RR^f^ late responders	95% CI	
		n	%^d^	n	%^e^	n	%^e^	n	%^e^					p-value^f^	
*Gender*														<0.001	
	Male	1315	56.5	455	34.6	396	30.1	464	35.3	1		1			
	Female	1013	43.5	475	46.9	275	27.1	263	26.0	1.84	1.51–2.43	1.23	0.99–1.52		
*Age (years)*														<0.001	
	< 20	468	20.1	179	38,2	116	24,8	173	37,0	1		1			
	20–29	1144	49.1	424	37,1	365	31,9	355	31,0	1.15	0.90–1.49	1.53	1.16–2.02		
	30–39	564	24.2	258	45,7	151	26,8	155	27,5	1.61	1.21–2.15	1.45	1.05–2.01		
	≥ 40	152	6.5	69	45,4	39	25,7	44	28,9	1.52	0.98–2.33	1.32	0.81–2.16		
*Language region of Switzerland*														0.011	
	German	1684	72.3	710	42,2	469	27,9	505	30,0	1		1			
	French	561	24.1	189	33,7	178	31,7	194	34,6	0.69	0.55–0.87	0.99	0.78–1.25		
	Italian	83	3.6	31	37,3	24	28,9	28	33,7	0.79	0.47–1.33	0.92	0.53–1.61		
*Nationality*														<0.001	
	Swiss	2017	89.3	868	43,0	602	29,8	547	27,1	1		1			
	German, Austrian, French, Italian^g^	102	4.5	35	34,3	28	27,5	39	38,2	0.57	0.35–0.90	0.65	0.40–1.07		
	Other	140	6.2	26	18,6	41	29,3	73	52,1	0.22	0.14–0.36	0.51	0.34–0.76		
*Neighborhood index of SEP*														0.754	
	First tertile (lowest SEP)	678	33.4	258	38,1	207	30,5	213	31,4	1		1			
	Second tertile	678	33.4	278	41,0	186	27,4	214	31,6	1.07	0.83–1.38	0.89	0.68–1.18		
	Third tertile (highest SEP)	677	33.3	265	39,1	198	29,2	214	31,6	1.02	0.79–1.32	0.95	0.73–1.25		
*Diagnosis (ICCC-3)*														0.020	
	I Leukemia	784	33.7	343	43,8	231	29,5	210	26,8	1		1			
	II Lymphoma	441	18.9	154	34,9	137	31,1	150	34,0	0.63	0.47–0.83	0.83	0.62–1.12		
	III CNS tumor	326	14.0	128	39,3	83	25,5	115	35,3	0.68	0.50–0.92	0.66	0.47–0.92		
	IV Neuroblastoma	102	4.4	41	40,2	27	26,5	34	33,3	0.74	0.45–1.20	0.72	0.42–1.24		
	V Retinoblastoma	57	2.5	19	33,3	18	31,6	20	35,1	0.58	0.30–1.11	0.82	0.42–1.59		
	VI & VII Renal & hepatic tumor	152	6.5	69	45,4	49	32,2	34	22,4	1.24	0.80–1.94	1.31	0.81–2.12		
	VIII Bone tumor	108	4.6	45	41,7	33	30,6	30	27,8	0.92	0.56–1.50	1.00	0.59–1.70		
	IX Soft tissue sarcoma	132	5.7	52	39,4	37	28,0	43	32,6	0.74	0.48–1.15	0.78	0.48–1.26		
	X Germ cell tumor	75	3.2	28	37,3	17	22,7	30	40,0	0.57	0.33–0.98	0.52	0.28–0.96		
	XI & XII Other tumor	41	1.7	10	24,4	11	26,8	20	48,8	0.31	0.14–0.67	0.50	0.23–1.07		
	Langerhans cell histiocytosis	110	4.7	41	37,3	28	25,5	41	37,3	0.61	0.38–0.98	0.62	0.37–1.04		
*Treatment*														0.041	
	Surgery only	289	12.6	110	38,1	66	22,8	113	39,1	0.69	0.51–0.92	0.56	0.40–0.78		
	Chemotherapy^h^	1126	49.2	461	40,9	340	30,2	325	28,9	1		1			
	Radiotherapy^i^	778	34.0	312	40,1	232	29,8	234	30,1	0.94	0.75–1.17	0.95	0.75–1.20		
	Bone marrow transplantation	95	4.2	41	43,2	27	28,4	27	28,4	1.07	0.64–1.78	0.96	0.55–1.67		
*Relapse*														0.540	
	No	2033	87.3	819	40,3	587	28,9	627	30,8	1					
	Yes	295	12.7	111	37,6	84	28,5	100	33,9	0.85	0.64–1.14	0.90	0.66–1.22		
*Age at diagnosis (years)*														0.719	
	< 5	841	36.1	346	41,1	234	27,8	261	31,0	1		1			
	5–9.9	635	27.3	241	38,0	195	30,7	199	31,3	0.91	0.71–1.17	1.09	0.84–1.42		
	≥ 10	852	36.6	343	40,3	242	28,4	267	31,3	0.97	0.77–1.22	1.01	0.79–1.30		
*Time since diagnosis (years)*														<0.001	
	< 10	240	10.3	84	35,0	58	24,2	98	40,8	1		1			
	10–19.9	1025	44.0	380	37,1	308	30,0	337	32,9	0.76	0.55–1.05	0.65	0.45–0.93		
	20–29.9	817	35.1	352	43,1	242	29,6	223	27,3	1.40	1.12–1.75	1.19	0.94–1.51		
	≥ 30	246	10.6	114	46,3	63	25,6	69	28,0	1.47	1.05–2.04	1.00	0.69–1.45		

^a^ Survivors responding to the first questionnaire sent (40.0%).

^b^ Survivors responding to the first reminder letter or second reminder phone call (28.8%).

^c^ Survivors not responding or declining participation (31.2%).

^d^ Column percenteges are given.

^e^ Row percentages are given.

^f^ RRs and p-values from univariable multinomial regression models comparing early and late responders with nonresponders.

^g^ Questionnaires were available in their mother languages (German, French, Italian).

^h^ Chemotherapy may include surgery.

^i^ Radiotherapy may include chemotherapy or surgery.

Abbreviations: CI, confidence interval; CNS, central nervous system; ICCC-3, International Classification of Childhood Cancer, third edition; n, number; RR, risk ratio; SEP, socioeconomic position.

### Estimation of potential nonresponse bias in our study

**Fig 2:**
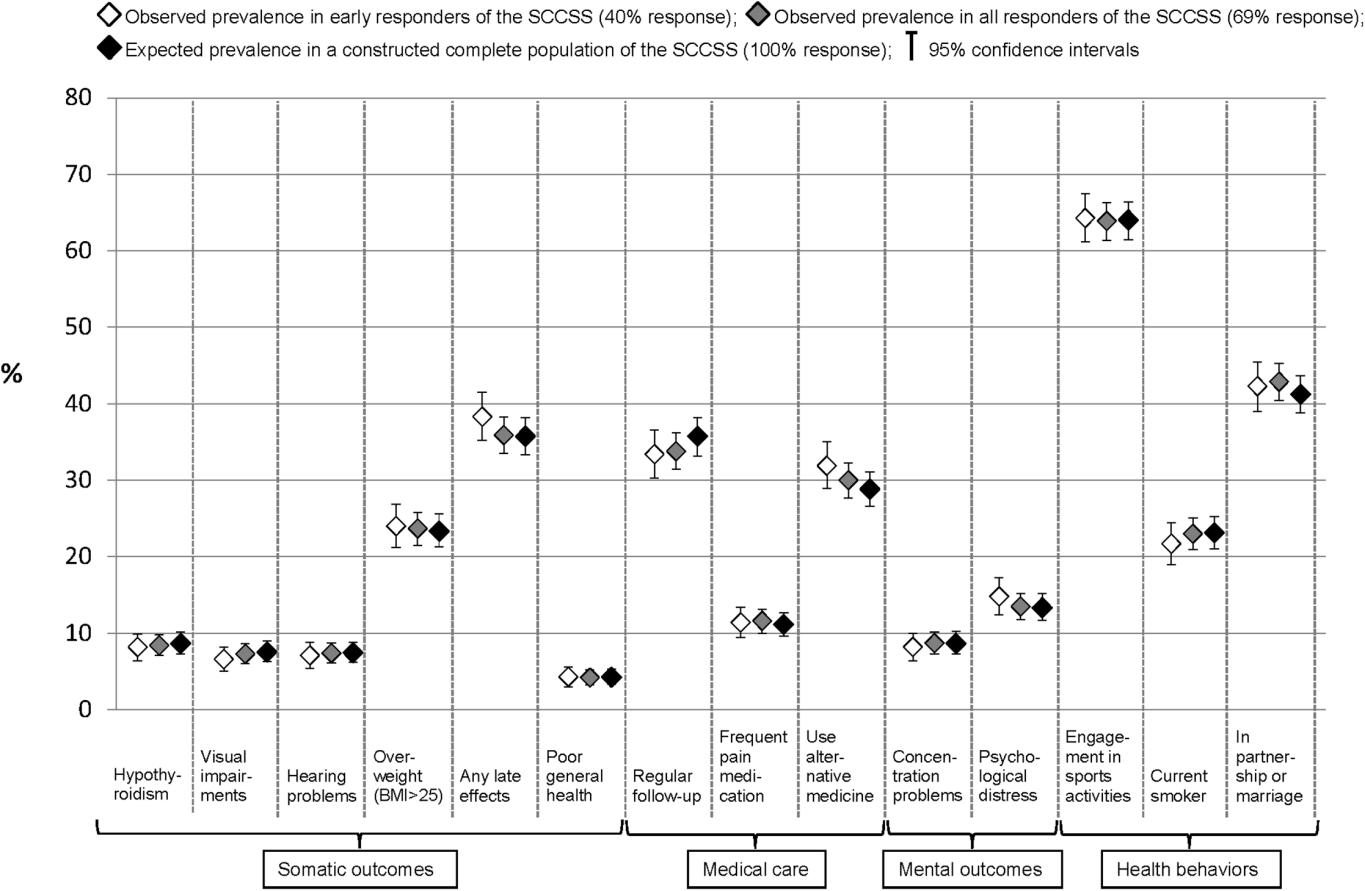
Comparison of self-reported outcomes between early responders, late responders, and a constructed complete population of the Swiss Childhood Cancer Survivor Study. Proportions and 95% confidence intervals for typical self-reported outcomes for somatic health, medical care, mental health, and health behaviors, comparing the observed prevalence from early responders (designated with a white diamond) and all responders (designated with a grey diamond) of the SCCSS with the expected prevalence in the representative complete population (designated with a black diamond). We constructed the complete population with inverse probability of participation weights (S1 File) (22). S1 Table describes how each of the outcomes compared was assessed and classified. Abbreviations: BMI, Body Mass Index; SCCSS, Swiss Childhood Cancer Survivor Study.

To estimate nonresponse bias and the gain from sending out additional reminder letters, we compared the prevalence of selected outcomes (S1 Table) of our questionnaire between the group of early responders (40%), all responders (69%), and the representative total population (100%, Fig 2 and Table 2). The prevalence estimates we observed in both samples of responders did not differ much in any clinically relevant way from either each other or the estimates we would expect in the total population. Early responders reported slightly more late effects (38.3%, 95% CI 35.2%-41.5%) than the group of all responders (35.9%, CI 33.5%-38.3%), and, than expected in the total population (35.7%, CI 33.3%-38.2%). Early responders used alternative medicine slightly more often (31.9%, CI 28.9%-35.0%) than all responders (30.0%, CI 27.7%-32.3%) or the total population (28.8%, CI 26.6%-31.1%). But responders were less likely to be in regular follow-up (33.4% of early responders, CI 30.3%-36.6%; 33.8% of all responders, CI 31.4%-36.2%) than expected in the total population (35.7%, CI 33.2%-38.2%). All other estimates were substantially similar in the three groups.

**Table 2:** Comparison of self-reported outcomes between early responders, late responders, and a representative complete population of the Swiss Childhood Cancer Survivor Study (description of the numbers presented in Fig 2).

		Early responders^a^ (n = 930)		All responders^b^ (n = 1601)		Complete population^c^ (n = 2328)	
		%	95% CI	%	95% CI	%	95% CI
*Somatic outcomes*							
	Hypothyroidism	8.2	6.4–9.9	8.4	7.1–9.8	8.6	7.3–10.1
	Visual impairments	6.6	5.0–8.2	7.3	6.0–8.6	7.5	6.3–9.0
	Hearing problems	7.1	5.4–8.8	7.4	6.1–8.7	7.4	6.2–8.8
	Overweight (BMI >25 kg/m^2^)	24.0	21.2–26.9	23.7	21.5–25.8	23.3	21.3–25.6
	Any late effects	38.3	35.2–41.5	35.9	33.5–38.3	35.7	33.3–38.2
	Poor general health	4.3	3.0–5.6	4.2	3.2–5.2	4.2	3.3–5.3
*Medical care*							
	Regular follow-up	33.4	30.3–36.6	33.8	31.4–36.2	35.7	33.2–38.2
	Frequent pain medication	11.4	9.4–13.4	11.6	10.0–13.1	11.1	9.6–12.7
	Use alternative medicine	31.9	28.9–35.0	30.0	27.7–32.3	28.8	26.6–31.1
*Mental outcomes*							
	Concentration problems	8.2	6.4–10.0	8.7	7.3–10.1	8.6	7.3–10.2
	Psychological distress	14.8	12.4–17.2	15.5	11.8–15.2	13.3	11.7–15.2
*Health behaviors*							
	Engagement in sports activities	64.3	61.2–67.5	63.9	61.4–66.3	64.0	61.5–66.4
	Current smoker	21.7	19.0–24.4	23.0	20.9–25.1	23.1	21.0–25.3
	In partnership or marriage	42.3	39.0–45.5	42.9	40.4–45.3	41.2	38.8–43.7

^a^ Survivors who responded to the initial questionnaire (40%).

^b^ All survivors who responded (69%).

^c^ Constructed complete population of the SCCSS (100%).

Abbreviations: BMI, body mass index; CI, confidence interval.

## Discussion

This is to our knowledge the first study to estimate the effect of nonresponse bias in a questionnaire-based study of childhood cancer survivors. We found differences in gender, language, nationality, diagnosis, and treatment between the groups of early responders, late responders, and nonresponders, with late responders falling between early responders and nonresponders for most characteristics. Despite this, the observed prevalence of specific outcomes in early responders and all responders was very close to the expected prevalence in a constructed complete population. This suggests that nonresponse bias plays a minor role in this survey, and did not result in over- or underestimation of the prevalence of the outcomes we studied.

### Limitations and strengths

Although we included many different variables to construct a population representative of a 100% response rate, there might be other unmeasured factors associated with participation in the questionnaire survey and outcomes of interest. For example, we could not include current health status, which might be relevant for this particular population of childhood cancer survivors. This could be a problem regarding the assumption underlying this analysis: specific subgroups of responders are representative for all eligible participants who share the same characteristics. In other words, we assume that the outcome in a specific substratum of participants—for example, females, aged >40 years, with low education, who suffered from CNS tumor and were treated with radiotherapy, without relapse—is observed at random. This assumption would not hold in some cases. For instance, it is possible that survivors with severe late effects and chronic health conditions were the least likely to respond to the survey and we could not adjust for this unmeasured factor. But since late effects correlate with type of diagnosis, treatment, and relapse, for which we did adjust, we do not think that such residual confounding is of great relevance in this study. Furthermore, we did not find that late responders reported more health problems than early responders (Fig 2 and Table 2).

Although we found no evidence of relevant response bias in the outcomes presented, all of which were selected a priori and represented different domains of the questionnaire, we cannot rule out the possibility that prevalence estimates of other outcomes might be biased [8]. The studied estimates are mainly related to health and treatment and might not be generalizable to other different outcomes. The same is true for the generalization of our results to other populations, cohorts or countries because response rates of a survey are specific to the population under study and influenced by various partly unknown factors (such as cultural and social factors of a society).

Finally, we assessed bias in prevalence and not in measures of associations such as odds ratios or hazard ratios. However, if the underlying distribution of characteristics is unbiased, the resulting measures of association also should be unbiased.

Our study has several strengths. First, our sampling frame was the national Swiss Childhood Cancer Registry, which includes all 5-year survivors in Switzerland and makes this study representative of all childhood cancer survivors in Switzerland. Second, the detailed information collected on the entire population in the SCCR allowed us to compare a variety of characteristics among responders and nonresponders that include demographic, socioeconomic, and clinical data. Such variables are known predictors of participation in a survey [5, 7, 10, 11], and are also commonly used to predict late outcomes in childhood cancer survivors [22–27]. Third, we had detailed information on every step of the survey, saved in a separate study database, allowing us to calculate scenarios of response behavior: we could compare participants who answered the initial questionnaire to all responders. This allowed us to appraise the gain of additional reminder efforts.

### Comparison with other studies and interpretation of results

We know of two methodological studies on selection bias or response behavior in childhood cancer survivors. Like ours, a 2010 study based on a request for a buccal-cell specimen found that participants were more likely to be female and nonimmigrants, though it did not estimate the extent of possible nonresponse bias [28]. Another recent study assessed potential bias from nonparticipation in a clinical cohort of long-term childhood cancer survivors and compared characteristics of participants with the whole source population [29]. They found similar frequencies for most characteristics with modest differences for sex, income, home-value, and urbanity. However, that study did not estimate the potential impact these differences may have had on prevalence estimates.

Similar to other studies on nonresponse bias conducted in the general population, we found that women and nonimmigrants were more likely to respond [5–7, 11, 28]. Unlike others, however, we did not find that socioeconomic position (SEP) measured at the neighborhood-level [6, 7, 10, 11] was associated with nonresponse. Our results might have been different if we had been able to use individual-based SEP measures such as education. Although we provided questionnaires and letters in French and Italian, survivors in the French- and Italian-speaking parts of Switzerland were less likely to respond. These survivors were perhaps less inclined to respond to a survey sent by a study center in the German-speaking part of Switzerland. Immigrants from Germany, Austria, France, and Italy were also less likely to respond. It is possible that they were not as interested in participating in a Swiss study; or, they might have had problems understanding the long and time-consuming questionnaire.

We were surprised to find that older survivors and those with longer intervals since diagnosis were more interested in participating in the survey. We anticipated that the greater the time since diagnosis, the greater the number of survivors who might lose interest. It is useful to know that this is not the case when planning studies on the growing population of very-long-term survivors of childhood cancer [30].

### Implications for practice

We found that follow-up reminders are effective. After an initial postal inquiry, a further postal and then a telephone reminder increased the overall response rate from 40% to 69%. However, the additional responses did not change the prevalence of the outcomes of interest we analyzed.

This phenomenon has been observed previously in telephone-based surveys that compared early with late responders [9, 12]. A methodological study has also explained why nonresponse does not necessarily bias the results of a survey [8]: every person is potentially either a responder or a nonresponder, depending on various characteristics and circumstances, and a nonresponse bias may cancel out across subgroups [8, 31]. For example, a woman would be more likely to respond to a survey, but if she is a migrant this factor is leveled off. This is useful information for survey planners: if a budget is too low to sustain reminders that would lead to a high response rate from a study population, results from the initial responders may still be sufficiently representative. However, it would be helpful to test this directly in a study population of interest by, for example, sending reminder letters to a random sample of nonresponders and analyzing the responses.

Our description of survivors who were less likely to respond may be of use as well. Some study aspects could be modified to solicit response from populations normally less likely to return questionnaires. For example, if questionnaires can be translated into additional languages, responses from immigrants might improve [32]. If citizenship influences response, it might be helpful to inform participants that their reply matters whether they are citizens of the country or not. Availability of online questionnaires and invitations by e-mail or mobile phone text messages might increase the response of participants aged <20 years [33, 34]. The many nonresponders who had only surgery might not see themselves as cancer survivors. A more generally formulated information letter could help ensure that those survivors feel they are part of the study population.

Our most important finding is that the observed prevalence of specific outcomes in the subgroup of early responders and the group of all responders differed little or not at all from the expected prevalences in the total population we constructed, which indicates no nonresponse bias. The underlying true prevalences were therefore neither over- nor underestimated. This is reassuring, when we interpret results from the SCCSS, and particularly important because results from cancer survivorship studies are commonly applied in clinical practice such as follow-up care of survivors or interventions to increase healthy behavior or quality of life [14, 26, 27, 35–39]. Based on the presented data we can be confident that the survey findings reflect the situation of all survivors in Switzerland.

## Conclusion

In our questionnaire survey, phone and mail reminders substantially increased the response rate. Early responders differed in several sociodemographic and clinical aspects from nonresponders, with late responders lying in between. But when we compared observed prevalence in respondents to expected prevalence in a constructed total population we found no evidence of relevant nonresponse bias in any of the outcomes we scrutinized. Results derived from the Swiss Childhood Cancer Survivors Study can therefore be generalized with some confidence to the corresponding population of cancer survivors in Switzerland and applied in clinical practice.

## Supporting information

S1 File The rational for the use of inverse probability of participation weights. (DOC)

S1 Table Questions and classification of typical outcomes of each section of the Swiss Childhood Cancer Survivor Study Questionnaire. (DOCX)

S2 Table Characteristics of survivors by type of response; risk ratios from multivariable multinomial logistic regression model. (DOCX)
