# Supplementary material for: No evidence of response bias in a population-based childhood cancer survivor questionnaire survey — Results from the Swiss Childhood Cancer Survivor Study
Source: PLoS One. 2017 May 2;12(5):e0176442. doi: 10.1371/journal.pone.0176442 (PMC5413049; doi:10.1371/journal.pone.0176442)
Supplement: S1 File — (DOC) [file pone.0176442.s001.doc]

**Supplemental Appendix I. The rationale for the use of inverse probability of participation weights**

The following table shows a simplified invented situation in the Swiss Childhood Cancer Survivor Study (SCCSS) with one dichotomous predictive factor (gender) for a possible prevalence estimate (smoking).

|  | Number of survivors in the total population of the SCCSS | Survivors who responded to the survey | Number of current smokers in those who responded | Percent of current smokers in those who responded | Probability to respond to the survey | Inverse of the probability to respond to the survey |
| --- | --- | --- | --- | --- | --- | --- |
| Female | 400 | 360 | 72 | 20% | 90.0% | 1.11 |
| Male | 600 | 300 | 150 | 50% | 50.0% | 2.00 |
| Total | 1000 | 660 | 222 | 34% |  |  |

We have 1000 eligible survivors of the SCCSS and 660 (66%) who responded to the survey with information on current smoking status. However, the response rate was not the same in males and females. A questionnaire was available for 90% of females and 50% of males. Among responders, 222 were current smokers (34%), 72 females (20%), and 150 males (50%). Clearly, it would be inappropriate to say that this observed prevalence of 34% of current smokers reflects the true prevalence of smoking among all 1000 survivors.

*Obtaining a corrected prevalence estimate of smoking:*

If we assume that for each gender the responders are representative of all the survivors of that gender, we can do the following calculation for the prevalence of smoking among all 1000 survivors. We expect to have 20% of smokers among all 400 women (80 expected to be current smokers) and 50% of smokers in all 600 men (300 expected to be current smoker). In total we expect 380 smokers among the 1000 survivors, i.e. a prevalence of 38%.

Mathematically, we get exactly the same result (38%) if we conducted a weighted analysis restricted to the 660 responders, using gender specific weights that are 1.11 and 2, derived as the inverse of the probability to have responded to the survey. This is what we called an analysis using *inverse probability of participation weights*. The advantages of the weighted approach are twofold. First, it can easily be extended to more than one predictive factor of a prevalence estimate using multivariable logistic regression. Second, in almost all statistical software it is possible to conduct an analysis in which the units of observation have differing statistical weight, and to obtain estimates and 95% confidence intervals that account for the weighting. However, we need to remember and acknowledge the assumption that all relevant predictive factors have been included in calculating the weights. The corrected prevalence estimate may still be biased, if this assumption does not hold. This is known as the assumption of no unmeasured confounding variables.
