# Supplementary material for: No evidence of response bias in a population-based childhood cancer survivor questionnaire survey — Results from the Swiss Childhood Cancer Survivor Study
Source: PLoS One. 2017 May 2;12(5):e0176442. doi: 10.1371/journal.pone.0176442 (PMC5413049; doi:10.1371/journal.pone.0176442)
Supplement: S1 Table — (DOCX) [file pone.0176442.s002.docx]

**S1 Table. Questions and classification of typical outcomes of each section of the Swiss Childhood Cancer Survivor Study Questionnaire**

|  | Name | Question | Answer categories | Classification |
| --- | --- | --- | --- | --- |
| **Somatic outcomes** | **Hypothyroidism** | Underfunction of the thyroid gland | 0 = No, currently not present  1 = Yes, currently present | 0 = No hypothyroidism  1 = Hypothyroidism |
|  | **Visual impairments** | Severe visual impairment or blind (on one or both eyes) | 0 = No, currently not present  1 = Yes, currently present | 0 = No visual impairments  1 = Visual impairments |
|  | **Hearing problems** | Problems with hearing | 0 = No, currently not present  1 = Yes, currently present | 0 = No hearing problems  1 = Hearing problems |
|  | **Overweight** | How tall are you? How much do you weight? |  | Body mass index ≤25kg/m^2^ = No overweight  Body mass index >25kg/m^2^ = Overweight |
|  | **Any late effects** | Do you suffer from late-effects of your former cancer disease? | 0 = No  1 = Yes | 0 = No late effects  1 = Any late effects |
|  | **Poor general health** | How would you rate your general health status? | 1 = Excellent  2 = Very good  3 = Good  4 = Not so good  5 = Poor | 1-3 = Good general health  4-5 = Poor general health |
| **Medical care** | **Regular follow-up** | Do you still have regular follow-up care for your former cancer disease? | 1 = Yes, I have regular follow-up care in my former cancer clinic  2 = Yes, I have regular follow-up care in another clinic or with another doctor  3 = No, my regular follow-up care is terminated but I sometimes visit a doctor  4 = No, my regular follow-up care is terminated and I haven’t seen a doctor for a long time | 3-4 = No regular follow-up  1-2 = Regular follow-up |
|  | **Frequent pain medication** | Medication against pain and fever? | 1 = Daily  2 = Several times per week  3 = Once a week  4 = Less than once a week | 4 = No frequent pain medication  1-3 = Frequent pain medication |
|  | **Use alternative medicine** | Do you use alternative medicine? | 0 = No  1 = Yes | 0 = No use of alternative medicine  1 = Use of alternative medicine |
| **Mental outcomes** | **Concentration problems** | Are you able to concentrate at school/at work? | 1 = Excellent  2 = Very good  3 = Good  4 = Not so good  5 = Bad | 1-3 = No concentration problems  4-5 = Concentration problems |
|  | **Psychological distress** | Brief Symptom Inventory (BSI) 18 |  | Transformed T-score <57 = No psychological distress  Transformed T-score ≥57 = Psychological distress |
| **Health behaviors** | **Engagement in sports activities** | Do you engage in gymnastics, fitness, or sports? | 0 = No  1 = Yes | 0 = No engagement in sports activities  1 = Engagement in sports activities |
|  | **Current smoker** | Have you ever smoked cigarettes? | 0 = No, I never smoked cigarettes  1 = Yes, I smoked before but I am not smoking anymore  2 = Yes, I am currently smoking | 0-1 = No current smoker  2 = Current smoker |
|  | **In partnership or marriage** | Do you live in a partnership or marriage? | 0 = No  1 = Yes | 0 = No partnership or marriage  1 = In a partnership or marriage |
