# Supplementary material for: No evidence of response bias in a population-based childhood cancer survivor questionnaire survey — Results from the Swiss Childhood Cancer Survivor Study
Source: PLoS One. 2017 May 2;12(5):e0176442. doi: 10.1371/journal.pone.0176442 (PMC5413049; doi:10.1371/journal.pone.0176442)
Supplement: S2 Table — (DOCX) [file pone.0176442.s003.docx]

**S2 Table. Characteristics of survivors by type of response; risk ratios from multivariable multinomial logistic regression model**

|  | | **Total**  **(n=2328)** | |  | **Early responders^a^**  **(n=930)** | |  | **Late responders^b^**  **(n=671)** | |  | **Non-responders^c^**  **(n=727)** | |  | **RR^d^ early responders** | **95% CI** |  | **RR^d^ late responders** | **95% CI** |  |  |
| --- | --- | --- | --- | --- | --- | --- | --- | --- | --- | --- | --- | --- | --- | --- | --- | --- | --- | --- | --- | --- |
|  | | **n** | **%** |  | **n** | **%** |  | **n** | **%** |  | **n** | **%** |  |  |  |  |  |  | **p-value^d^** | |
| *Gender* | |  |  |  |  |  |  |  |  |  |  |  |  |  |  |  |  |  | <0.001 | |
|  | Male | 1315 | 56.5 |  | 455 | 48.9 |  | 396 | 59.0 |  | 464 | 63.8 |  | 1 |  |  | 1 |  |  | |
|  | Female | 1013 | 43.5 |  | 475 | 51.1 |  | 275 | 41.0 |  | 263 | 36.2 |  | 1.94 | 1.57-2.41 |  | 1.29 | 1.02-1.62 |  | |
| *Age (years)* | |  |  |  |  |  |  |  |  |  |  |  |  |  |  |  |  |  | 0.008 | |
|  | < 20 | 468 | 20.1 |  | 179 | 19.3 |  | 116 | 17.3 |  | 173 | 23.8 |  | 1 |  |  | 1 |  |  | |
|  | 20 - 29 | 1144 | 49.1 |  | 424 | 45.6 |  | 365 | 54.4 |  | 355 | 48.8 |  | 1.14 | 0.85-1.53 |  | 1.54 | 1.12-2.11 |  | |
|  | 30 - 39 | 564 | 24.2 |  | 258 | 27.7 |  | 151 | 22.5 |  | 155 | 21.3 |  | 1.81 | 1.17-2.80 |  | 1.68 | 1.05-2.69 |  | |
|  | ≥ 40 | 152 | 6.5 |  | 69 | 7.4 |  | 39 | 5.8 |  | 44 | 6.1 |  | 1.71 | 0.86-3.40 |  | 1.52 | 0.72-3.18 |  | |
| *Language region of Switzerland* | |  |  |  |  |  |  |  |  |  |  |  |  |  |  |  |  |  | 0.053 | |
|  | German | 1684 | 72.3 |  | 710 | 76.4 |  | 469 | 69.9 |  | 505 | 69.5 |  | 1 |  |  | 1 |  |  | |
|  | French | 561 | 24.1 |  | 189 | 20.3 |  | 178 | 26.5 |  | 194 | 26.7 |  | 0.72 | 0.56-0.92 |  | 0.99 | 0.77-1.28 |  | |
|  | Italian | 83 | 3.6 |  | 31 | 3.3 |  | 24 | 3.6 |  | 28 | 3.9 |  | 0.83 | 0.48-1.46 |  | 0.92 | 0.51-1.65 |  | |
| *Nationality* | |  |  |  |  |  |  |  |  |  |  |  |  |  |  |  |  |  | <0.001 | |
|  | Swiss | 2017 | 89.3 |  | 868 | 93.4 |  | 602 | 89.7 |  | 547 | 83.0 |  | 1 |  |  | 1 |  |  | |
|  | German, Austrian, French, Italian^e^ | 102 | 4.5 |  | 35 | 3.8 |  | 28 | 4.2 |  | 39 | 5.9 |  | 0.53 | 0.33-0.86 |  | 0.64 | 0.39-1.07 |  | |
|  | Other | 140 | 6.2 |  | 26 | 2.8 |  | 41 | 6.1 |  | 73 | 11.1 |  | 0.26 | 0.16-0.42 |  | 0.55 | 0.36-0.84 |  | |
| *Neighborhood index of SEP* | |  |  |  |  |  |  |  |  |  |  |  |  |  |  |  |  |  |  | |
|  | First tertile (lowest SEP) | 678 | 33.4 |  | 258 | 32.2 |  | 207 | 35.0 |  | 213 | 33.2 |  | n.a.^f^ |  |  |  |  |  | |
|  | Second tertile | 678 | 33.4 |  | 278 | 34.7 |  | 186 | 31.5 |  | 214 | 33.4 |  |  |  |  |  |  |  | |
|  | Third tertile (highest SEP) | 677 | 33.3 |  | 265 | 33.1 |  | 198 | 33.5 |  | 214 | 33.4 |  |  |  |  |  |  |  | |
| *Diagnosis (ICCC-3)* | |  |  |  |  |  |  |  |  |  |  |  |  |  |  |  |  |  | 0.599 | |
|  | I Leukemia | 784 | 33.7 |  | 343 | 36.9 |  | 231 | 34.4 |  | 210 | 28.9 |  | 1 |  |  | 1 |  |  | |
|  | II Lymphoma | 441 | 18.9 |  | 154 | 16.6 |  | 137 | 20.4 |  | 150 | 20.6 |  | 0.66 | 0.48-0.91 |  | 0.83 | 0.60-1.16 |  | |
|  | III CNS tumor | 326 | 14.0 |  | 128 | 13.8 |  | 83 | 12.4 |  | 115 | 15.8 |  | 0.81 | 0.55-1.21 |  | 0.86 | 0.56-1.31 |  | |
|  | IV Neuroblastoma | 102 | 4.4 |  | 41 | 4.4 |  | 27 | 4.0 |  | 34 | 4.7 |  | 0.85 | 0.50-1.47 |  | 0.81 | 0.45-1.47 |  | |
|  | V Retinoblastoma | 57 | 2.5 |  | 19 | 2.0 |  | 18 | 2.7 |  | 20 | 2.8 |  | 0.66 | 0.32-1.36 |  | 0.98 | 0.47-2.03 |  | |
|  | VI & VII Renal & hepatic tumor | 152 | 6.5 |  | 69 | 7.4 |  | 49 | 7.3 |  | 34 | 4.7 |  | 1.27 | 0.78-2.06 |  | 1.32 | 0.79-2.19 |  | |
|  | VIII Bone tumor | 108 | 4.6 |  | 45 | 4.8 |  | 33 | 4.9 |  | 30 | 4.1 |  | 1.02 | 0.59-1.75 |  | 1.07 | 0.60-1.88 |  | |
|  | IX Soft tissue sarcoma | 132 | 5.7 |  | 52 | 5.6 |  | 37 | 5.5 |  | 43 | 5.9 |  | 0.77 | 0.47-1.25 |  | 0.80 | 0.48-1.34 |  | |
|  | X Germ cell tumor | 75 | 3.2 |  | 28 | 3.0 |  | 17 | 2.5 |  | 30 | 4.1 |  | 0.53 | 0.29-0.95 |  | 0.55 | 0.28-1.05 |  | |
|  | XI & XII Other tumor | 41 | 1.7 |  | 10 | 1.1 |  | 11 | 1.6 |  | 20 | 2.8 |  | 0.50 | 0.20-1.20 |  | 0.80 | 0.34-1.90 |  | |
|  | Langerhans cell histiocytosis | 110 | 4.7 |  | 41 | 4.4 |  | 28 | 4.2 |  | 41 | 5.6 |  | 0.69 | 0.40-1.19 |  | 0.76 | 0.42-1.36 |  | |
| *Treatment* | |  |  |  |  |  |  |  |  |  |  |  |  |  |  |  |  |  | 0.527 | |
|  | Surgery only | 289 | 12.6 |  | 110 | 11.9 |  | 66 | 9.9 |  | 113 | 16.2 |  | 0.86 | 0.58-1.29 |  | 0.64 | 0.41-1.00 |  | |
|  | Chemotherapy^g^ | 1126 | 49.2 |  | 461 | 49.8 |  | 340 | 51.1 |  | 325 | 46.5 |  | 1 |  |  | 1 |  |  | |
|  | Radiotherapy^h^ | 778 | 34.0 |  | 312 | 33.8 |  | 232 | 34.9 |  | 234 | 33.5 |  | 0.94 | 0.72-1.22 |  | 0.97 | 0.74-1.28 |  | |
|  | Bone marrow transplantation | 95 | 4.2 |  | 41 | 4.4 |  | 27 | 4.1 |  | 27 | 3.9 |  | 1.13 | 0.66-1.96 |  | 1.00 | 0.56-1.79 |  | |
| *Relapse* | |  |  |  |  |  |  |  |  |  |  |  |  |  |  |  |  |  |  | |
|  | No | 2033 | 87.3 |  | 819 | 88.1 |  | 587 | 87.5 |  | 627 | 86.2 |  | n.a.^f^ |  |  |  |  |  | |
|  | Yes | 295 | 12.7 |  | 111 | 11.9 |  | 84 | 12.5 |  | 100 | 13.8 |  |  |  |  |  |  |  | |
| *Age at diagnosis (years)* | |  |  |  |  |  |  |  |  |  |  |  |  |  |  |  |  |  |  | |
|  | < 5 | 841 | 36.1 |  | 364 | 37.2 |  | 234 | 34.9 |  | 261 | 35.9 |  | n.a.^f^ |  |  |  |  |  | |
|  | 5 - 9.9 | 635 | 27.3 |  | 241 | 25.9 |  | 195 | 29.0 |  | 199 | 27.4 |  |  |  |  |  |  |  | |
|  | ≥ 10 | 852 | 36.6 |  | 343 | 36.9 |  | 242 | 36.1 |  | 267 | 36.7 |  |  |  |  |  |  |  | |
| *Time since diagnosis (years)* | |  |  |  |  |  |  |  |  |  |  |  |  |  |  |  |  |  | 0.432 | |
|  | < 10 | 240 | 10.3 |  | 84 | 9.0 |  | 58 | 8.6 |  | 98 | 13.5 |  | 1 |  |  | 1 |  |  | |
|  | 10 - 19.9 | 1025 | 44.0 |  | 380 | 40.9 |  | 308 | 45.9 |  | 337 | 46.4 |  | 0.75 | 0.53-1.07 |  | 0.66 | 0.45-0.97 |  | |
|  | 20 - 29.9 | 817 | 35.1 |  | 352 | 37.9 |  | 242 | 36.1 |  | 223 | 30.7 |  | 1.12 | 0.83-1.51 |  | 1.01 | 0.74-1.38 |  | |
|  | ≥ 30 | 246 | 10.6 |  | 114 | 12.3 |  | 63 | 9.4 |  | 69 | 9.5 |  | 1.18 | 0.68-2.04 |  | 1.03 | 0.57-1.85 |  | |

^a^ Survivors who responded to the initial questionnaire sent (40.0%).

^b^ Survivors who responded only after reminding them (28.8%).

^c^ Survivors who did not respond at all (31.2%).

^d^ RRs and p-values from a multivariable multinomial regression model comparing early and late responders with non-responders.

^e^ Questionnaires were available in their mother languages (German, French, Italian).

^f^ Factors not associated in the univariable regression models were excluded from the multivariable model.

^g^ Chemotherapy may include surgery.

^h^ Radiotherapy may include chemotherapy or surgery.

Abbreviations: CI, Confidence Interval; CNS, Central Nervous System; ICCC-3, International Classification of Childhood Cancer – third edition; n.a., not applicable; n, number; RR, Risk Ratio; SEP, Socio-Economic Position.
